# 

**Published:** 1874-07

**Authors:** 


### Books and Pamphlets Received.

A Treatise on Food and Dietetics Physiologically and Therapeutically Considered. By F. W. Pavy, M. D., F. R. S. Philadelphia: Henry C. Lea, 1874. Buffalo: T. Butler & Son.

A Conspectus of The Medical Sciences; Comprising Manuals of Anatomy,

Physiology, Chemistry, Materia Medica, Practice of Medicine, Surgery and Obstetrics. By Henry Hartshorne, A. M., M. D. Philadelphia: Henry C. Lea, 1874. Buffalo: T. Butler & Son.

Electro-Therapeutics, a Condensed Manual of Medical Electricity. By D. F. Lincoln, M. D. Philadelphia: Henry C. Lea, 1874. Buffalo: T. Butler & Son.

Report of the Quebec Lunatic Asylum addressed to the Honorable the Prime Minister of the Province of Quebec. By the Medical Superintendents. Quebec: L. H. Huot, 1873.

Transactions of the Kentucky State Medical Society for 1874. Louisville: J. P. Morton & Co., 1874.

Proceeding of the Royal Society of London. Nos. 145, 146 and 147.

Atmospheric Electricity and Ozone. Their Relation to Health and Disease. By Geo. M. Beard, M. D. From Popular Science Monthly, Feb., 1874.

A New method of Treating Malignant Tumours by Electrolyzing the Base. By Geo. M. Beard, M. D. From Archives of Electrology and Neurology, May, 1874.

Transactions of the Medical Society of the District of Columbia, July, 1874.

Recent Advances in the Diagnosis of Diseases of the Nervous System. By H. R. Bigelow, M. D., Hartford, Conn. From Detroit Review of Medicine and Pharmacy.

Rare Cases of Congenital Syphilis. By L. Duncan Bulkley, A. M., M. D. From New York Medical Journal, May, 1874.

